# Comparison of Hydralazine and Labetalol to lower severe hypertension in pregnancy

**DOI:** 10.12669/pjms.332.12243

**Published:** 2017

**Authors:** Ayesha Khan, Sajida Hafeez, Farah Deeba Nasrullah

**Affiliations:** 1 Prof. Ayesha Khan, FRCOG. Department of Obstetrics & Gynaecology, Civil Hospital, Karachi, Dow University of Health Sciences, Karachi, Pakistan; 2 Dr. Sajida Hafeez, FCPS. Department of Obstetrics & Gynaecology, Civil Hospital, Karachi, Dow University of Health Sciences, Karachi, Pakistan; 3 Dr. Farah Deeba Nasrullah, FCPS. Department of Obstetrics & Gynaecology, Civil Hospital, Karachi, Dow University of Health Sciences, Karachi, Pakistan

**Keywords:** Hydralazine, Labetalol, Mean arterial pressures, Pregnancy induced hypertension

## Abstract

**Objective:**

To compare the intravenous Labetalol versus intravenous hydralazine in patients having severe Pregnancy induced hypertension (PIH) and pre eclampsia (PE) in pregnancy.

**Methods:**

Seventy eight women admitted in the Department of Gynecology and Obstetrics, Civil Hospital Karachi, having severe PIH/PE and fulfilling the inclusion criteria were included in the study. Random selection of patients was performed using sealed opaqe envelop for administration of either intravenous noted (IV) Labetalol or Hydrallazine. The mean fall in the MAP in each group was noted. This data was analyzed by applying SPSS version 13. The study was conducted from November 2012 to April 2013.

**Results:**

The mean (±SD) age of the labetalol group was 27.46 (±5.28) years while that in the hydralazine group was 26.28 (±5.17) years. The mean fall in MAP observed in the labetalol group was 29.10 ± 7.21 mmHg and that in the hydralazine group was 25.05 ± 10.15 mmHg which was statistically significant with the p value being 0.046.

**Conclusion:**

Intra Venous labetalol lowered MAP more than hydralazine, when administered to pregnant females with severe Pregnancy induced hypertension and pre eclampsia in pregnancy.

## INTRODUCTION

Hypertensive disorders of pregnancy account for a leading cause of peripartum morbidity and mortality.^1^ The incidence of these disorders is 3-8% in developed countries.^2^,^3^ Incidence is 10-30 times among women of low socio economic status.^4^ The frequency was reported as 5.34% from Karachi^5^ and 3.2% from Lahore.^6^ These women are at higher risk of developing hypertension and its associated complications during pregnancy. Severe hypertension is a life threatening multisystem disease. A failure to treat hypertension promptly may result in placental abruption, eclampsia, HELLP syndrome pulmonary oedema and fetal demise.^7^,^8^ A prompt control of blood pressure is of crucial importance to prevent sequel of disease. In developing countries and certainly in the centre where study was conducted antenatal care is a neglected issue and is considered unnecessary. Frequently patients reach hospital or are referred in critical situation. In such instances a prompt treatment to deal with emergency is mandatory. Hence a parenteral drug appears to be a better choice.

Recent guidance from the National Institute of Health and Clinical Excellence, UK, recommends inpatient treatment of severe hypertension of pregnancy with labetalol (oral or intravenous), intravenous hydralazine or oral nifedipine as first line alternative antihypertensives within the critical care setting. For many years hydralazine has been the antihypertensive of choice for women with severe hypertension in pregnancy. Its use was associated with adverse effects. Labetalol is also used to treat acute hypertension in pregnancy as a first line drug. Neonatal bradycardia was the most associated adverse effect of concern

The rationale of the study was to compare the impact of intravenous labetalol and intravenous hydralazine in pregnant patients with severe hypertension in terms of reduction of mean arterial pressure as there is no significant data available in Pakistan on this topic. The results of the study might help to identify a drug which would be expected to give better results to avert maternal and fetal complications.

## METHODS

This comparative study was conducted in the department of Obstetrics and Gynecology Dow University of Health Sciences and Civil Hospital Karachi. To compare blood pressure lowering effect of labetalol and Hydralazine, two groups of women were identified, each group consisting of 39 women.

The sample size was calculated using formula n=2ZPQ/D. n=minimum sample size, Z=95% confidence interval using 1.96, P=prevalence of severe pre eclampsia 2, Q=1.0-p, D=degree of accuracy desired, usually set at 0.05, n=37 adding 5% attrition rate anticipated response rate 95%. The selected sample size was37/0.95= 38.9. Total of 39 women in each group were taken making a sample size of 78 was calculated.^9^

Non probability sampling technique was used for sample collection. Seventy eight women with pregnancy induced hypertension at a gestational age between 24-37 weeks with a Systolic Blood Pressure of >160mm and Diastolic Blood Pressure > 110 mm were considered fulfilling inclusion criteria and were recruited for study. Pregnant women with medical disorders, situations where these drugs were contra indicated, women with severe bradycardia, history of reaction to these drugs were excluded from the study.

**Table-I:** Age distribution.

*Age Of patients (Years)*	*Labetalol Group*		*Hydralazine Group*		*Total*	

	*(n=39)*	*%*	*(n=39)*	*%*	*(n=78)*	*%*
15-25	13	33.3	21	53.8	34	43.6
26-35	24	61.5	17	43.6	41	52.6
>35	02	05.1	01	02.6	03	03.8

Overall Mean age (±S.D) = 26.87 (±5.22) Years. Mean age (±S.D) (Labetalol) = 27.46 (±5.28) Year Mean age (±S.D) (Hydralazine) = 26.28 (±5.17) Years.

### Data collection procedure

Total 78 patients who were admitted in the Obstetrics and Gynecology Department Unit II of Civil Hospital Karachi, and fulfilled the inclusion criteria were enrolled in the study after taking informed consent. A detailed history was taken and the gestational age of the patient was calculated using date of last menstrual period. The patient’s blood pressure was then recorded. With patient sitting comfortably blood pressure was recorded using Mercury monometer. An appropriate cuff size was used. Reading of Systolic and Diastolic blood pressure were taken at Korat coff 1 and V respectively.

The patients were then randomly assigned to either of the two groups that is the Labetalol group or the Hydralazine group using sealed opaque envelope method. Once the group of the patient was assigned, an intravenous access was established using cannula of 18 gauge and medication was administered to each group.

### Labetalol Group

In this group, 20mg intravenous slow bolus dose of labetalol was administered followed by 40mg if not effective within 20 minutes, followed by 80mg every 20 minutes to a maximum of three doses.

### Hydralazine Group

In this group, 5mg of hydralazine as a slow bolus was given intravenously, and repeated every 20 minutes until the desired effect was achieved or maximum of three doses. All patients were monitored actively during the whole process including repeated blood pressure readings after every 15 minutes and the general status too was taken in account.

**Table-II:** Parity Status.

*Parity*	*Labetalol*		*Hydralazine Group*		*Total*	

	*(n= 39*)	*%*	*(n= 39)*	*%*	*(n=78)*	*%*
<3	29	74.4	31	79.5	60	76.9
>3	10	25.6	08	20.5	18	23.1

Overall Mean Parity (±S.D) = 1.94 (±1.95) Mean Parity (±S.D) (labetalol) = 1.92(±1.82) Mean Parity (±S.D) (Hydralazine) = 1.95 (±2.10)

Data were entered on a pre-designed performa. Confounding variables were controlled by excluding those patients who had specific contraindications to either labetalol or hydralazine, or had a known allergy to either of the two drugs. All patients who suffered from severe bradycardia were also excluded.

**Table-III:** Gestational Age.

*Gestational age*	*Labetalol Group*		*Hydralazine Group*		*Total*	

	(n=39)	%	(n=39)	%	(n=78)	%
<31 weeks	10	25.6	10	25.6	20	25.6
>31 weeks	29	74.4	29	74.4	58	74.4

Overall Mean Gestational Age (±S.D) =33.10 (±2.60) weeks Mean Gestational Age (±S.D) (Labetalol) = 32.23 (±2.44) weeks Mean Gestational Age (±S.D) (Hydralazine)=32.97 (±2.78) weeks

### Data analysis

The software program SPSS for Windows version 13 (SPSS Incorporated, Chicago, Illinois, USA) was utilized for all statistical analyses. Frequencies and percentages were used to summarize age, parity, and gestational age. Mean ± standard deviation (SD) were computed for numerical variables like age distribution, gestational age, SBP, DBP, number of doses of drugs used and MAP. Stratification was done with regards to age, parity, and gestational age to observe the effects of these on outcomes.

## RESULTS

The mean age of patients in labetalol group *was 27*.46 years while in hydralazine group it was 26.28 years. The mean parity in labetalol group was 1.92 and hydralazine group was1.95. Mean gestational age in labetalol group was 33.23 and mean gestational age in hydralazine group was 32.97.

Out of 78 patients, 44 (56.4%) required more than one dose of the drug used. Single dose of labetalol was sufficient in reducing the mean arterial pressure (MAP) to the desired level in 20 (51.3%) of the patients. In contrast single dose was sufficient only in 14 (35.9%) patients in the hydralazine group. The mean (±SD) number of doses required in the labetalol group was 1.59 (±0.68) while in the hydralazine group number of doses required was 1.90 (±0.79)

**Table-IV:** SBP, DBP and MAP at presentation (n=78).

*Group*	*SBP*	*DBP*	*MAP*
Hydralazine (n=39)	172.31±12.24	116.15±5.90	134.46±6.35

Value are given in Mean±SD

At admission of patients the Systolic blood pressure (SBP) in the labetalol group was 172.69±14.1 mmHg while in the hydralazine group it was 172.31±12.24 mmHg. The diastolic blood pressure (DBP) at presentation was 116.67±5.78 mmHg in the labetalol group and 116.15 ± 5.90 mmHg in the hydralazine group. Hence the mean MAP in the former was 134.95 ± 7.10 mmHg and 134.46±6.35 mmHg in the later group.

**Table-V:** SBP, DBP AND MAP after treatment (n=78).

*Group*	*SBP*	*DBP*	*MAP*
Labetalol (n=39)	140.49±8.88	91.03±7.88	107.10±7.19
Hydralazine (n=39)	141.03±8.59	94.49±7.05	109.57±7.00

Values are given in Mean±SD

After treatment the mean SBP in the labetalol group was 140.49±8.88 mmHg while in the hydralazine group it was 141.03±8.59 mmHg. The DBP after treatment was 91.03±7.88 mmHg in the labetalol group and 94.49±7.05 mmHg in the hydralazine group. Hence the mean MAP in the former was 107.10±7.19 mmHg and 109.54±7.00 mmHg in the later group.

The mean fall in MAP observed in the labetalol group was 29.10 ±7.21 mmHg and that in the hydralazine group was 25.05±10.15 mmHg. On application of independent t test the difference in the fall of MAP in these two groups was statistically significant with the p value being 0.046.

Patients in Hydrallazine group had headache and tachycardia more often as compare to women in Labetalol group. Side effects like maternal hypotension, nausea, vomiting, adverse fetal heart rate recording were not noted significantly in either group. Low Apgar score at 5 minutes was not noted which could be attributed to use of these drugs

## DISCUSSION

Hypertension in pregnancy contributes significantly towards the maternal morbidity and mortality in developed as well as developing world. The gravity of the condition is pronounced in cases of severe hypertension. There is consensus that sustained severe hypertension in pregnancy should be treated as it is considered to be a risk factor for maternal end organ complications such as cerebral stroke. Threshold of blood pressure at which treatment should start is important. At the same time treatment should not cause a marked lowering of blood pressure leading to reduction in utero placental circulation. In a case series Martin et al found 96% of women had a Systolic Blood Pressure of 160mm Hg or above immediately prior to stroke and only 13% had Diastolic Blood pressure of 110 mm Hg or above.^10^ Martin study was criticized because of small sample size making use of statistical model impossible yet a Systolic Blood pressure threshold of 160 mm Hg or above appear significant.^11^ A confidential enquiry into Maternal and Child Health attributed fetal intracranial hemorrhage a result of inadequate treatment of severe Systolic Blood Pressure of 160mmHg in women with pre eclampsia.^12^ National High Blood Pressure Program recommends a Systolic Blood Pressure of 160mmHg or above and a Diastolic Blood Pressure 110mmHg threshold to start treatment.^13^

**Table-VI:** Fall in mean arterial pressure (n=78).

	*Labetalol*	*Hydralazine*
Fall in MAP (Mean±SD)	29.10±7.21	25.05±10.15

P value = 0.046 Calculated by application of independent t-test.

American College of Obstetricians and Gynecologist recommends parenteral labetalol and hydralazine as first line drug for the treatment of acute severe hypertension.^14^ Hydralazine has been serving as anti-hypertensive since over 40 years. It acts as a vasodilator, decreases peripheral resistance and lowers blood pressure. The effects are of short duration and system reset to the blood pressure levels necessary to maintain pressure in kidney necessary for natriuresis. It is not used as primary drug because it elicits a reflex sympathetic stimulation of heart which would results in increased heart rate and cardiac output and risk of angina with myocardial infarction.

Labetalol is a non-selective beta blocker and post synaptic alpha-1blocking agent. Labetalol may be considered as first line drug, but there is a potential risk of fetal bradycardia.

Both drugs have been used extensively in the management of pregnant women with higher blood pressure. Trivedi Swati at el. in their comparative study on Labetalol and Hydrallazine found both drugs as effective and quick acting antihypertensive agents in severe pre eclampsia.^15^ Numba in his study demonstrated Labetalol and Hydralazine effective and rapid anti-hypertensive agent in hypertension crises.^9^ The time taken to lower blood pressure and number of doses of Hydralazine and Labetalol were similar in Numba study. A study from Delgado De Rasquel also reported similar findings.^16^ In the current study the mean number of doses in Labetalol group was 1.59 and in Hydralazine group it was 1.90 doses.

In a study conducted by Mable et al authors found hydralazine lowered mean arterial pressure more than Labetalol that is 13.3 versus 11.2mm Hg.^17^ A study by Ashe et al showed comparable results.^18^ A Cochrane review failed to judge superiority of the hydralazine or labetalol. It concluded that the evidence is insufficient to decide superiority of Hydralazine or Labetalol.^19^

Contrary to above findings, in the current study reduction of blood pressure with labetalol was significant with p value of 0.046. This differs from the study of Mable and Ashe.^17^,^18^ The difference could be due to the fact that current study had a calculated sample size which was comparatively larger. Vigil V Gracia failed to exhibit superiority of hydralazine over labetalol.^20^ Authors concluded no statistically significant difference between two drugs raising the question about the choice of hydralazine as first line drug. A study on post natal hypertensive patients failed to observe any statistically significant difference between hydralazine and labetalol.^21^

Magee in his meta-analysis proposed labetalol as a promising alternate as first line agent.^22^ The author also expressed concerns regarding safety profile of hydralazine. In Cochrane review meta-analysis data from the two drugs was not significant to decide superiority of one drug over another

Studies reporting the efficacy of hydralazine and labetalol as anti-hypertensive mostly favored null hypothesis demonstrating no superiority of one drug over another in achieving blood pressure reduction. The current study has reported better results in reduction of blood pressure with labetalol as compared to hydralazine. This study contain a sample size of 78 with 39 in each group. A well designed, randomized controlled trial with adequate sample size will help to determine better drug for control of hypertension particularly in context of Pakistani pregnant population.

### Limitations of the study

Blood Pressure recordings were not maintained on study proforma after two blood pressure readings. Although American College of Obstetricians and Gynaecologist recommends 10 mg dose of parenteral Hydrllazine, a dose of 5mg of Hydrallazine was preferred due to the fact that women from study population were not over weight.

## CONCLUSION

Our results have shown better control of blood pressure with labetalol. Administration of drug during pregnancy and more so in cases of critical situation needs care full judgment. A balance between safety profile of drug and a given dose is of crucial importance. Therefore until conclusive results are obtained regarding superiority of hydralazine or labetalol, choice of drug should be on clinicians experience and discretion.

### Authors` Contribution

**AK:** Supervised study, wrote manuscript and is responsible for intellectual integrity of the study.

**SH:** Conducted study, analyzed results.

**FDN:** Arranged references.

## References

[1] Arulkumaran N, Lightsone L (2013). Severe pre-eclampsia and hypertensive crises. Best Pract Res Clin Obstet Gynecol.

[2] Carty DM, Delles C, Dominiczak AF (2010). Pre-eclampsia and future maternal health. J Hyperetens.

[3] Khalil AA, Cooper DJ, Harrington KF (2009). Pulse wave analysis:a preliminary study of a novel technique for the prediction of pre-eclampsia. BJOG.

[4] Duley L (2009). The global impact of pre-eclampsia and eclampsia. Semin perinatal.

[5] Hossin N, Shah N, Khan N, Lata S, Khan NH (2011). Maternal and perinatal outcome of hypertensive disorder of pregnancy at Tertiary Care Hospital. JHUHS.

[6] Khawaja NP, Parveen A, Hussain U, Zahid B, Rehman R Frequency and Obstetric outcome of hypertensive disorders of pregnancy.

[7] Sibai B, Dekkar G, Kupferminc M (2005). Pre-eclampsia. Lancet.

[8] Hauth JC, Ewell MG, Levine RJ, Esterlitz JR, Sibai B, Curet LB (2000). Pregnancy outcomes in healthy nulliparas who developed hypertension. Calcium for Preeclampsia Prevention Study Group. Obstet Gynecol.

[9] Nombur LI, Agida ET, Isah AY, Ekele BA (2014). A comparison of Hydrallazine and Labetalol in the management of severe pre eclampsia. J Womens Health Care.

[10] Martin JN, Thigpen BD, Moore RC, Rose CH, Cushman J, May W (2005). Stroke and severe pre-eclampsia and eclampsia:a paradigm shift focusing on systolic blood pressure. Obstet Gynecol.

[11] Magee LA, Abalos E, von Dadelszen P, Sibai B, Easterling T, Walkinshaw S (2011). CHIPS Study Group. How to manage hypertension in pregnancy effectively. Br J Clin Pharmacol.

[12] Raheem IA, Saaid R, Omar SZ, Tan PC (2012). Oral nifedipine versus intravenous labetalol for acute blood pressure control in hypertensive emergencies of pregnancy:a randomized trial. BJOG.

[13] (2000). Report of the National High Blood Pressure Education Program Working Group on High Blood Pressure pregnancy. Am J Obstet Gynecol.

[14] (2011). The American College of Obstetricians and Gynecologists. Emergent therapy for acute-onset, severe hypertension with preeclampsia or eclampsia. Committee opinion.

[15] Swati T, Lila V, Lata R, Prachi G, Pratibha A, Tuhar P (2016). A comparative study of itravenous Labetalol and intravenous Hydrallazine on mean arterial blood pressure changes in pregnant women with hypertensive emergency. Sch J App Med Sci.

[16] Delgado De Pasquale S, Velarde R, Rayes O, De La Ossa K (2014). Hydrallazine vs Labetalol for the treatment of severe hypertensive disorders of pregnancy;A randomised controlled trial. Pregnancy Hypertens.

[17] Mable WC, Gonzalez AR, Sibai BM, Amon E (1987). A comparative trial of labetalol and hydralazine in the acute management of severe hypertension complicating pregnancy. Obstet Gynecol.

[18] Ashe RG, Moodley J, Richards AM, Philpott RH (1987). Comparison of labetalol and dihydralazine of pregnancy. S Afr Med J.

[19] Duley L, Henderson-Smart DJ, Meher S (2006). Drugs for the treatment of very high blood pressure during pregnancy. Cochrane Database Syst Rev.

[20] Vigil-De Gracia P, Lasso M, Ruiz E, Vega-Malek JC, de Mena FT (2006). Severe hypertension in pregnancy:hydralazine or labetalol. A randomized clinical trial. Eur J Obstet Gynecol Reprod Biol.

[21] Vigil-De Gracia P, Ruiz E, Lopez JC, de Jaramillo IA, Vega-Maleck JC, Pinzon J (2007). Management of severe hypertension in the post partum period with intravenous hydrallazineor labetalol:A randomized clinical trial. Hypertens Pregnancy.

[22] Magee LA, Cham C, Waterman EJ, Ohlsson A, von Dadelszen P (2003). Hydralazine for treatment of severe hypertension in pregnancy:meta-analysis. Br Med J.

